# Low Caregiver-Child Positive Escalation in Childhood Predicts Late Adolescent BPD

**DOI:** 10.1007/s10802-026-01513-3

**Published:** 2026-10-14

**Authors:** Charlie Huntington, Kirsten Gilbert, Alecia C. Vogel, Rebecca Tillman, Brittany Haus, Deanna M. Barch, Joan L. Luby, Diana J. Whalen

**Affiliations:** 1 Department of Psychiatry, Washington University in St. Louis, St. Louis, MO USA; 2 Department of Psychological and Brain Sciences, Washington University in St. Louis, St. Louis, MO USA; 3 Department of Radiology, Washington University in St. Louis, St. Louis, MO USA

**Keywords:** Borderline personality disorder, Major depressive disorder, Observational data, Positive escalation, Developmental

## Abstract

Borderline personality disorder (BPD) and major depressive disorder (MDD) are highly comorbid, yet little is known about developmental family processes that distinguish between individuals who will go on to develop MDD versus BPD. Using longitudinal, observational data from the Preschool Depression Study, we tested whether caregiver behaviors and caregiver-child interaction patterns during a conflict discussion task predicted later BPD vs. MDD symptoms. Caregiver-child dyads (*N* = 161) completed five-minute conflict discussions at up to three timepoints across late childhood and early adolescence; these discussions were observationally coded for caregiver and dyadic behaviors. Outcomes were self-reported BPD symptoms and clinical interview-based MDD symptoms assessed in late adolescence. After controlling for caregiver BPD and MDD and correcting for the false discovery rate, hierarchical linear regression models showed that lower dyadic positive escalation between caregiver and child predicted elevated BPD symptoms in late adolescence. Associations with MDD symptoms were not significant. These findings suggest that maintaining or building positive affect during challenging caregiver-child interactions may be a protective interpersonal process that is more specific to risk for BPD than risk for MDD. Implications for our understanding of the development of BPD versus MDD in childhood and adolescence are discussed.

Borderline personality disorder (BPD) is a debilitating psychiatric disorder characterized by substantial difficulties with emotion regulation and interpersonal functioning, instability in sense of self, and suicidality and self-injurious behaviors (American Psychiatric Association, 2022). Per Linehan (1993)’s Biosocial Theory, BPD emerges as an individual’s innate biological vulnerability to emotion dysregulation interacts with an invalidating social environment. More specifically, social environments that invalidate children’s private emotional experiences are thought to reinforce extremes of emotional expression (i.e., alternating between inhibiting and expressing emotion), leaving children with few resources to effectively handle distress, especially in interpersonal contexts (Crowell et al., 2009).

As Linehan (1993) proposed a developmental model for the etiology of BPD, it is unsurprising that much of the research on invalidating environments has focused on the context of the family (Grove & Crowell, 2019), and parent–child interactions more specifically (Musser et al., 2018). Harsh and punitive parenting styles (Beeney et al., 2021; Franssens et al., 2021; Hallquist et al., 2015), as well as specific behaviors such as poor parental self-control, parental negative emotionality, emotional withdrawal, overinvolvement, invalidation, and inconsistent or chaotic behaviors (Boucher et al., 2017; Hallquist et al., 2015), are predictive of children’s BPD symptoms. Experiencing child maltreatment from parents is also strongly implicated in subsequent BPD symptomatology (Ibrahim et al., 2018). In alignment with biosocial theory, parents’ unsupportive responses to children’s negative emotions are particularly implicated in BPD risk (Musser et al., 2018; Whalen et al., 2014).

This caretaking pathway is understood to be transactional, rather than unidirectional; a child’s predisposition toward emotional lability may also provoke less effective parenting responses (Stepp et al., 2014). Parents of individuals with BPD describe their children as overly sensitive or possessing a difficult temperament (Boucher et al., 2017). Impulsivity, reactivity, and dysregulation in children predict less effective parenting practices over time (Crowell et al., 2009; Yan et al., 2021), such as more negative and fewer positive responses (Milan & Carlone, 2018), leading to lower quality interactions and relationships over time (Boucher et al., 2017). In this way, parent–child interactions may engender development of the maladaptive traits that characterize BPD (De Fruyt & De Clercq, 2014). Moreover, there is some evidence that effective parenting practices and dyadic processes may be protective against BPD risk (Franssens et al., 2021; Whalen et al., 2014). More supportive and involved parenting behaviors, such as maternal positive affect and validation, predict fewer BPD symptoms in both at-risk (Whalen et al., 2014) and community samples of preteens (Franssens et al., 2021; Whalen et al., 2014).

At the same time, individuals with BPD experience high rates of comorbidity with other psychiatric disorders, making it difficult to discern whether parent and parent–child behaviors demonstrate specificity in increasing BPD risk relative to other disorders, or might be implicated in a range of negative psychopathological outcomes for offspring. Comorbidity of BPD with mood disorders is especially high (Zimmerman & Morgan, 2013); most individuals diagnosed with BPD will at some point also be diagnosed with major depressive disorder (MDD) (Gunderson et al., 2008; Shah & Zanarini, 2018). This is unsurprising, as several traits – affective instability, suicidality, low mood and feelings of worthlessness – characterize both disorders (Kew et al., 2025). It has been posited that early mood symptoms may lead to an eventual MDD diagnosis – an instance of homotypic continuity – while for other individuals, early mood symptoms may predict later receiving a different diagnosis, such as BPD (i.e., heterotypic continuity; Zarrella et al., 2017). Given the significant distinctions between prognosis and treatment approaches to MDD versus BPD (Rao & Broadbear, 2019), identifying precursors of one diagnostic pathway versus the other is critical to providing effective support. This is especially the case because, although mood symptoms are present in both disorders, MDD severity appears to be heightened in individuals with BPD relative to those without (Skodol et al., 2011), underscoring the need for more research into their shared versus individual predictors.

To date, such literature is limited and mostly points to a pattern of multifinality (Cicchetti & Rogosch, 1996), wherein the same early risk factors implicated in the development of BPD are also implicated in other disorders, such as MDD (Beeney et al., 2021; Stepp et al., 2016). However, potential differences in trajectories toward BPD versus MDD have been identified. Specifically, Beeney and colleagues (2021) observed that among adolescent girls, poorer behavioral control, harsh punishment from parents, and more relational aggression at a younger age were more predictive of subsequent BPD than MDD, while greater loneliness, life stress, and poorer adolescent-caregiver trust more strongly predicted MDD than BPD. The present study aims to build on these findings by incorporating observational data of caregiver and caregiver-child behaviors, which avoid some of the limitations of self-report measures (Achenbach, 2006).

While parenting behaviors may individually or collectively affect BPD and MDD risk, these behaviors should be considered in the context of broader parent characteristics, including parental psychopathology. Children of mothers with BPD and/or MDD diagnoses are at increased risk of being diagnosed with the same disorder (Goodman et al., 2011). Maternal depression predicts child depressive symptoms as early as the toddler years (Gartstein & Bateman, 2008), and successful treatment of parents’ depression seems to improve the depressive symptoms of their children (Gunlicks & Weissman, 2008). There is also evidence that MDD and BPD predict parenting behaviors that have been associated with offspring MDD and BPD risk. Maternal BPD symptoms are associated with less sensitive and attuned and more controlling, hostile, and intrusive parenting (Eyden et al., 2016; Kiel et al., 2011; Mahan et al., 2018). Mothers with elevated BPD symptoms seem to inhibit autonomy in their adolescent children more than mothers without elevated BPD symptoms (Mahan et al., 2018). Meanwhile, depressed mothers are often less responsive, sensitive, and engaged with their children, and demonstrate more rigid structuring of interactions (Goodman & Garber, 2017; Paulson et al., 2006). Maternal depression is also associated with expressing more negative and less positive affect toward children (Dix & Meunier, 2009), as well as being more neglectful, more aggressive, and using more punishment in discipline (McCabe, 2014).

To date, the potential for parenting and parent–child behaviors to differentially predict subsequent risk for BPD versus MDD, despite their comorbidity, has received minimal attention. To the best of our knowledge, apart from the work by Beeney et al. (2021), parenting and parent–child behaviors have not been evaluated for their potential to predict BPD and MDD symptoms in children in the same study, nor have these longitudinal differences been evaluated using observational data. The current study leverages longitudinal observational data from caregiver-child interactions to investigate how caregiving and caregiver-child behaviors, in the context of caregiver psychopathology, might predict BPD versus MDD during late adolescence.

Observational studies of parent–child interactions have been used to understand parenting predictors of child BPD and MDD, but never to concurrently examine both disorders. A common paradigm in observational studies with adolescent or preadolescent children is the conflict discussion, wherein mother and child discuss a difficult topic in their relationship. Such research has found associations between offspring BPD symptoms and observed maternal insensitivity (Fleck et al., 2023), maternal disorganized-controlling behavior (Lyons-Ruth et al., 2013; Mahan et al., 2018), role confusion and negative escalation (Lyons-Ruth et al., 2015; Whalen et al., 2014), lower maternal structuring (Fleck et al., 2023), maternal hostility (Lyons-Ruth et al., 2013), and low maternal support/validation (Dixon-Gordon et al., 2016). Meanwhile, observational studies focused on MDD as an outcome have linked offspring MDD symptoms to less engaged parenting (Chapman et al., 2016), negative escalation (Yap et al., 2011), parental dismissal of a child’s anger (O’Neal et al., 2017), parental aggression (Schwartz et al., 2012, 2014; Sheeber et al., 2007), high rates of parental negative behaviors and low rates of positive behaviors and affect (McMakin et al., 2011; Schwartz et al., 2014), and parental suppression of adolescent positivity (Chapman et al., 2016) during conflict discussions. Broadly speaking, observational studies suggest both parenting and parent–child behaviors that could confer shared risk for MDD and BPD (e.g., negative escalation, parental negative affect), as well as behaviors more specific in conferring risk for MDD (e.g. less engagement; invalidation of positive emotion; reinforcement of negative child affect; low rates of positive parental behaviors) and BPD (e.g., role confusion in the dyad; intrusive, controlling, and disorganized parenting strategies; invalidation of negative affect).

Although a litany of caregiver behaviors and caregiver-child interactions have been identified as correlates of MDD and/or BPD risk in the literature, we focus our attention here on the subset regularly measured in observational studies. We used longitudinal data to test whether behaviors that theoretically confer shared risk and specific risk are in fact differentially versus mutually predictive of offspring MDD and/or BPD symptoms in late adolescence, as assessed through observation of caregiver-child interactions across late childhood and early adolescence. We hypothesized that caregiver negative affect, negative escalation, and positive escalation would confer shared risk, while negative extrinsic emotion regulation and dyadic role confusion would confer risk for BPD and caregiver positive affect would confer risk for MDD.

## Methods

### Participants

**Fig. 1:**
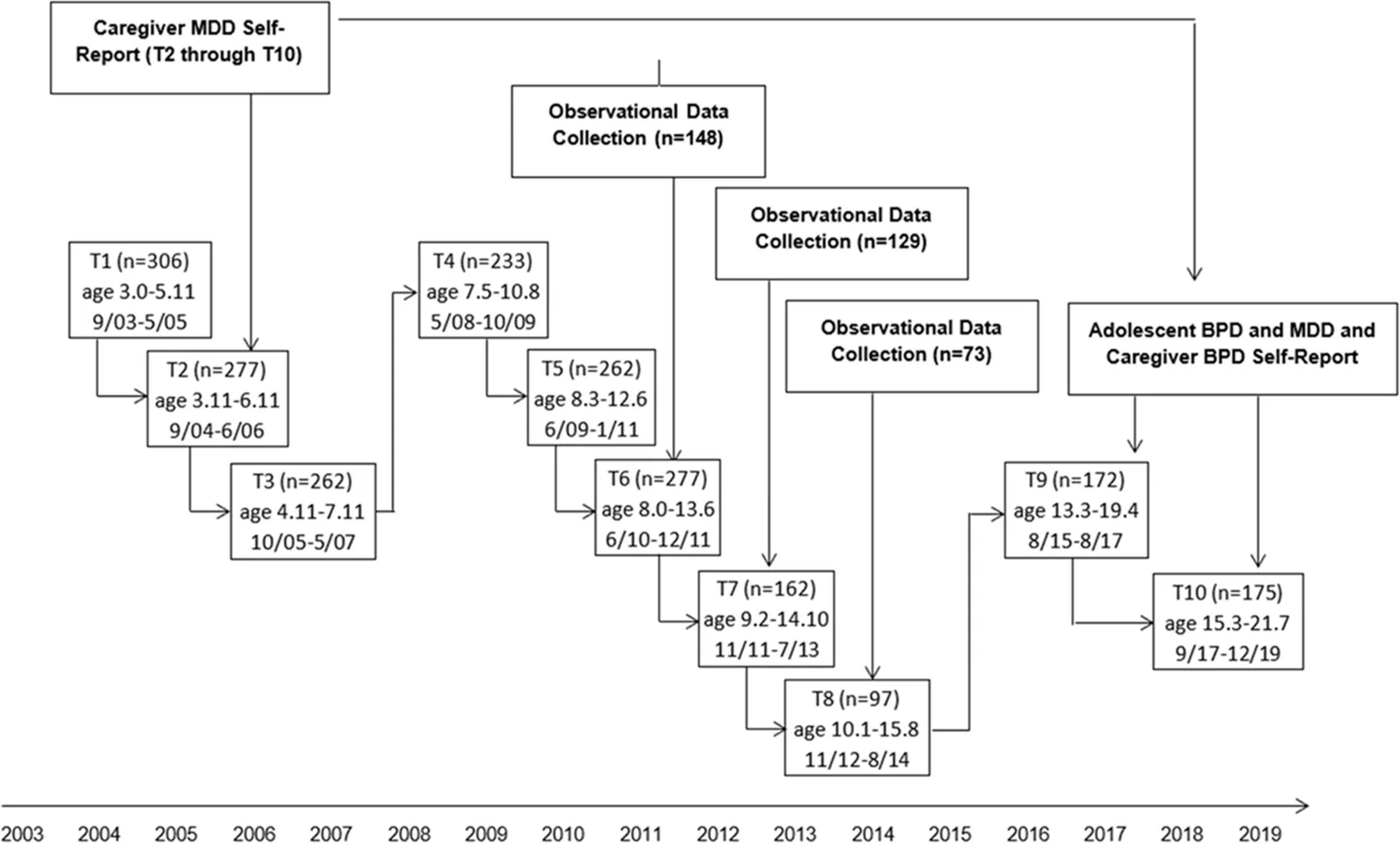
Preschool depression study (PDS) flowchart and observational data collection

Participants were 161 out of 306 children enrolled in the longitudinal Preschool Depression Study (see Luby et al., 2009) at the Early Emotional Development Program at Washington University School of Medicine in St. Louis. All study procedures were approved by the Institutional Review Board of the Washington University School of Medicine and parental written consent and child assent were obtained prior to participation. This phase of the study from which our outcomes are drawn, which followed the sample into adolescence and young adulthood, sought to prospectively investigate associations between childhood factors and later mental health outcomes. Participants were initially recruited during the preschool period (ages 3–6; *M* age 4.45, *SD* = 0.80) from daycare and primary care centers in the St. Louis metropolitan area and were selected after oversampling for depressive or disruptive behavior symptoms. These children returned for up to nine additional visits occurring 1–3 years apart (*M* age = 18.57, *SD* = 1.23 at last wave), including caregiver and child clinical interviews and questionnaires related to social and emotional functioning (see Fig. 1 for a flowchart of study visits). The subsample of participants included here completed at least one conflict discussion task with their caregiver during a study visit in late childhood/early adolescence and at least one assessment of BPD and MDD symptoms in later adolescence. Additionally, caregivers needed to complete measures of their own BPD and MDD symptoms at one or more study waves. For participants with inconsistent caregiver informants at different study waves (e.g. mother at some waves and father at others), care was taken to ensure all conflict discussion task and caregiver psychopathology data included in the analyses was obtained from a single caregiver. Three participants with all the required variables were excluded from analyses due to missing data when restricting to a single caregiver informant. An additional nine participants switched caregiver informants across study waves but were retained in the analyses after selecting a single caregiver (with the most complete data) and dropping data obtained at waves with a different caregiver informant. Caregivers were 94.4% biological mother, 4.4% biological father, 0.6% adoptive mother, and 0.6% grandmother.

### Measures

#### Observed Caregiver-Child Behaviors

At waves 6 through 8 (and only these waves), participants and their caregivers completed the ‘Hot Topics’ conflict discussion task (Hetherington et al., 1992) as part of a broader dyadic interaction protocol. Caregivers and adolescents first completed a 34-item questionnaire that asked them to identify topics they had discussed during the last 4 weeks. For the topics discussed, caregivers and adolescents then rated how they felt about the topic (from 1 [calm] to 5 [angry]). The research team then selected the 2 topics chosen by both reporters with the highest average score across both reporters on the calm-to-angry scale. The dyad was then asked to discuss the issues and attempt to reach a resolution for each during a five-minute videotaped conversation, which was subsequently coded by raters blind to diagnostic status.

The coding manual for the conflict discussion task was developed in two phases using a macro-behavioral observational approach (e.g. Prasad et al., 2023). In phase 1, trained research staff, individually and collectively, reviewed 20 caregiver-child interaction videos from the current study, drawing on the conflict discussion as well as other tasks, to identify self- and interpersonal-functioning indicators. Self-functioning (i.e., disturbances in identity and self-direction) and interpersonal functioning indicators were prioritized to align with the focus of the larger study on personality pathology, and the same manual and codes were used for all caregiver-child interactive tasks across all study waves. Following expert review and a confirmatory literature review, an initial draft manual was created, drawing on prior coding definitions for most behaviors and introducing some new codes. This manual was iteratively refined across several months by resolving wording and coding disagreements as the team reviewed additional tapes (see Supplemental Materials for a list of all codes). In phase 2, the coding team completed a structured training and ongoing calibration meetings to reach ≥ 70% agreement on new videos not used for the creation of the manual.

Coders were aware of the study’s general aims but were blind to the specific hypotheses and to key adolescent and caregiver characteristics (e.g. psychopathology ratings/diagnoses). For each video, coders rated child, caregiver, and dyadic behaviors. This study focuses specifically on a subset of caregiver and dyadic behavioral codes selected a priori based on prior literature linking these interaction patterns to risk for BPD and/or MDD. Each video was coded by one member of the coding team that consisted of nine coders. All coders had at least a bachelor’s degree in a relevant discipline (e.g. psychology), and several had received master’s degrees in a relevant discipline as well, including three licensed clinicians. In addition to coding the tasks included here, the coding team was also engaged in coding other caregiver-child interaction tasks conducted at earlier waves of this longitudinal study. The coding team met weekly for reliability training until reliability was met and maintained for several months, then met biweekly for the remainder of coding.

Interrater reliability was assessed using 152 of the 1438 videos coded across study waves 2 through 8, with between 1 and 8 coders rating each of the 152 videos, for a total of 900 coders’ ratings compared to the master coder’s ratings. When individual coders fell below acceptable reliability levels, the head of the coding team, a master’s level clinician, met with them individually and assigned them extra coding videos until reliability was re-established. Six codes – caregiver positive affect, caregiver negative affect, caregiver negative extrinsic emotion regulation, dyadic role confusion, dyadic positive escalation, dyadic negative escalation – were chosen based on prior literature connecting them to offspring MDD and/or BPD (see Table 1). Positive and negative affect were defined as positivity and negativity, respectively, of the caregiver’s vocal tone, facial expressions, and body language. Positive escalation was defined as one speaker responding to positivity from the other with positivity of their own, creating a snowball effect; negative escalation was defined the same way, but with exchange of negative affect. Negative extrinsic emotion regulation was defined as caregiver behaviors that, in seeking to emotionally regulate the child, also increase the child’s negative affect or decrease their positive affect, such as by invalidating their emotions. Finally, dyadic role confusion was defined as the extent to which the caregiver and child step outside of their roles as caregiver and child (e.g. with the child providing care or regulation for the caregiver).

**Table 1:** Definitions for observational codes used in analyses

Code	Definition	Previous observational findings
Caregiver positive affect	This code assessed the positiveness of the individual's tone of voice (e.g., bright or happy), facial expressions (e.g., smiling), and body language (e.g., open posture, laughter)	Negatively associated with child BPD (Whalen et al., 2014) and child MDD (McMakin et al., 2011; Schwartz et al., 2012, 2014)
Caregiver negative affect	This code assessed the overall level of negative affect (e.g., anger, frustration, tension, and irritation) expressed by the individual through tone of voice (e.g., cold, impatient, angry), facial expressions (e.g., frowning), and body language (e.g., constriction of the neck and shoulders)	Positively associated with offspring MDD (Schwartz et al., 2014) and BPD (Fleck et al., 2023; Whalen et al., 2014)
Caregiver negative extrinsic emotion regulation	This code captured parental behaviors, whether conscious or unconscious, that may increase the child’s negative affect or decrease positive affect. Examples include invalidating the child’s thoughts or emotions, blaming or criticizing the child, or explicitly downplaying something positive the child experienced or felt	Related constructs, such as maternal invalidation (Dixon-Gordon et al., 2016), maternal psychological control (Mahan et al., 2018), and maternal insensitivity (Williams et al., 2023) have been associated with offspring BPD
Dyadic role confusion	This is a global code indicating whether the parent and child act as parent and child, or demonstrate other dynamics, like parent as child or child as parent	Implicated in offspring BPD (Lyons-Ruth et al., 2015; Macfie et al., 2017)
Dyadic positive escalation	This measure rates how often positive behaviors of one partner are responded to with positive behaviors from the other partner, creating a snowball effect	Predicted lower BPD symptoms later in development (Whalen et al., 2014); associated with lower offspring MDD (McMakin et al., 2011)
Dyadic negative escalation	This measure rates how often negative behaviors of one partner are responded to with negative behaviors from the other partner, creating a snowball effect	Associated with greater offspring BPD (Lyons-Ruth et al., 2015; Whalen et al., 2014) and MDD (McMakin et al., 2011; Yap et al., 2011) symptoms

The codes for positive affect, negative affect, positive escalation, and negative escalation were based on the Revised Interactional Dimensions Coding System (Furman & Shomaker, 2008), as modified by Whalen et al. (2014). Negative extrinsic emotion regulation was derived from codes for negative discipline and intrusiveness in the Parent–Child Interaction Rating Scales (Brady-Smith et al., 1999). Dyadic role confusion was derived from prior coding of role confusion between caregiver and child (Zvara et al., 2018). Reliability was calculated for the conflict discussions across the three waves and all coders, with kappa values falling in the moderate range for all codes except for dyadic role confusion, which fell in the fair range (i.e. κ = 0.55 for caregiver positive affect, κ = 0.56 for caregiver negative affect, κ = 0.50 for caregiver negative extrinsic emotion regulation, κ = 0.31 for dyadic role confusion, κ = 0.43 for dyadic positive escalation, and κ = 0.61 for dyadic negative escalation; see Table 1 for code definitions).

Codes were rated on a 3-point scale (0 = none/low, 1 = some/moderate, or 2 = high/a lot). For caregiver positive affect, caregiver negative affect, and caregiver negative extrinsic emotion regulation, the mean score across waves was calculated for analysis. Due to low endorsement of dyadic role confusion, dyadic positive escalation, and dyadic negative escalation, codes of some/moderate and high/a lot were collapsed to create a dichotomous variable, and the maximum score across waves was used for analysis.

#### Adolescent BPD Symptoms

The Borderline Personality Features Scale for Children (BPFS-C; Crick et al., 2005) is a 24-item self-report questionnaire assessing symptoms of BPD. Items are scored on a 5-point Likert scale with values ranging from 1 = not at all true to 5 = always true, and the 24 items were summed for a total score. Four items (e.g., ‘I’m a pretty happy person’, ‘I feel pretty much the same way all the time. My feelings don’t change very often’) were reverse scored prior to calculating the total score. The BPFS-C was administered at waves 9 (Cronbach’s alpha = .90) and 10 (Cronbach’s alpha = .92), and the maximum score at those two waves was utilized as the BPD severity outcome measure.

#### Adolescent Depressive Symptoms

The Kiddie Schedule for Affective Disorders and Schizophrenia (KSADS; Kaufman et al., 1997) was administered separately to the participant and their caregiver at waves 9 (Cronbach’s alpha = .90) and 10 (Cronbach’s alpha = .88). Symptoms endorsed by participant and/or caregiver were considered present, and an MDD core score was calculated as the total number of the nine core MDD symptoms endorsed. The maximum MDD core score at the two waves was used as the MDD severity outcome in analyses.

#### Caregiver BPD Symptoms

The Personality Assessment Inventory – Borderline Scale (PAI-BOR; Morey, 1991), a 24-item self-report questionnaire assessing symptoms of BPD, was administered to the caregiver at waves 9 (Cronbach’s alpha = .90) and 10 (Cronbach’s alpha = .91). The items are scored on a 4-point Likert scale with values ranging from 0 = false/not at all true to 3 = very true, and the 24 items were summed for a total score. The maximum total score at waves 9 and 10 was used as a covariate in analyses.

#### Caregiver Depressive Symptoms

The Beck Depression Inventory – 2nd Edition (BDI-II; Beck et al., 1996), a 21-item self-report questionnaire assessing symptoms of depression, was administered to the caregiver at waves 2–10 (range of Cronbach’s alpha = .88—.94). Items were on a 0–3 scale and were summed for a total depression severity score, with higher scores indicating greater severity. The maximum score across all study waves was used as a covariate in analyses.

#### Childhood Depressive Symptoms

An MDD core score was calculated based on depression symptoms endorsed on the Preschool Age Psychiatric Assessment (PAPA) or Child and Adolescent Psychiatric Assessment (CAPA). The PAPA (Egger & Angold, 2004) was administered to the caregiver when children were aged 7 and younger. The CAPA (Angold & Costello, 2000) was used for subjects aged 8 and older, administered to only caregivers when participants were 8 years old and both caregiver and child participant when children were 9 years and older. Symptoms endorsed by participant and/or caregiver were considered present, and an MDD core score was calculated at each wave as the total number of the nine core MDD symptoms endorsed. The mean MDD core score across the 8 waves was used as the childhood MDD severity covariate in analyses. Cronbach’s alpha ranged from .52 to .64 across waves 1–8.

### Procedure

At each wave, participants and their caregivers completed interview and questionnaire measures. Child depressive symptoms were measured by caregiver report at waves 1 through 8, while adolescent depressive symptoms were measured by caregiver and adolescent report at waves 9 and 10. Caregivers reported their own depressive symptoms at waves 2 through 10. Caregiver and adolescent each reported on their own BPD symptoms at waves 9 and 10. Finally, participants and their caregivers completed the ‘Hot Topics’ conflict discussion task at waves 6 through 8. Participants were an average of 11.16 years old (SD = .88) at wave 6, when the first conflict discussions occurred, and an average of 16.27 years old (SD = 1.25) when BPD data were first collected at wave 9. Maximum scores across caregiver- and self-report measures were chosen to account for the episodic nature of depression (Van Eeden et al., 2019) and because BPD symptoms also show peaks in severity that may be best reflected by a maximum score (Choi-Kain et al., 2020).

### Statistical Analysis

Participant and caregiver BPD and MDD severity scores were checked for outlying values, and values more than 3 standard deviations from the mean were Winsorized by assigning the largest value within 3 standard deviations of the mean. For BPD severity, 1 participant value and 3 caregiver values were outliers, and for MDD severity, there were no participant outliers and only 1 caregiver outlier.

Hierarchical linear regression models were utilized to assess the association between Hot Topics codes and later BPD and MDD severity. Regardless of outcome, the Step 1 model included only the Hot Topics code predictor. For models with BPD severity as the outcome, Step 2 added in caregiver BPD severity, and Step 3 added in caregiver MDD severity as covariates. For models with MDD severity as the outcome, Step 2 added in caregiver MDD severity, and Step 3 added in caregiver BPD severity as covariates. Finally, for both the MDD and BPD models, Step 4 added biological sex and, to control for having oversampled for depressive symptoms, the average of participants’ core MDD symptoms across the first eight waves of the study (i.e. all data collected prior to the outcome data). False discovery rate (FDR) correction was applied across the six models at each step to account for multiple comparisons. Analyses were not pre-registered.

## Results

Table 2 provides descriptive statistics of participant characteristics, including demographics, outcome measures, Hot Topics codes, and covariates. In terms of biological sex, 50.3% of the participants were female and 49.7% were male. Roughly half (52.8%) of subjects were White, 36.0% were Black, and 11.2% were another race. At the final assessment point included in the study, participants were 18.25 years old on average (*SD* = 1.35). For the caregiver codes, scores fell below the midpoint of the 3-point scale from 0 to 2, indicating relatively low levels of positive affect (*M* = 0.72, *SD* = 0.62), negative affect (*M* = 0.41, *SD* = 0.47), and negative extrinsic emotion regulation (*M* = 0.51, *SD* = 0.57). As noted above, very few dyads were scored as ‘high/a lot’ on the dyadic codes, so these codes were recoded as dichotomous variables reflecting no versus any amount of behavior: dyadic role confusion, positive escalation, and negative escalation were observed in 23.6%, 41.0%, and 19.3% of dyads, respectively.

**Table 2:** Adolescent participant characteristics (*N* = 161)

Demographics/Covariates	%	n			
Female sex	50.3	81			
Race					
White	52.8	85			
Black	36.0	58			
Other	11.2	18			
	**Mean**	**SD**	**Minimum**	**Maximum**	Cronbach’s alphas**
Age at last assessment	18.25	1.35	14.34	21.63	–
Childhood MDD core*	2.07	2.70	0	8	0.52 – 0.64
Outcomes	**Mean**	**SD**	**Minimum**	**Maximum**	Cronbach’s alphas**
BPFS-C total score	59.95	14.24	27	94	0.90 – 0.92
MDD core score	2.07	2.70	0	9	0.88 – 0.90
Hot Topics Codes	**Mean**	**SD**	**Minimum**	**Maximum**	
Caregiver positive affect	0.72	0.62	0	2	
Caregiver negative affect	0.41	0.47	0	2	
Caregiver negative extrinsic emotion regulation	0.51	0.57	0	2	
	**%**	**n**			
Dyadic role confusion	23.6	38			
Dyadic positive escalation	41.0	66			
Dyadic negative escalation	19.3	31			
Caregiver BPD and MDD severity	**Mean**	**SD**	**Minimum**	**Maximum**	Cronbach’s alphas**
Caregiver PAI-BOR total	18.66	11.38	0	52	0.90 – 0.91
Caregiver BDI-II total	13.86	10.15	0	43	0.88 – 0.94

^*^Childhood MDD core = maximum of MDD core scores at waves 1–8; **Cronbach’s alphas calculated at each wave of administration

Table 3 contains bivariate correlations between all variables of interest. Dyadic positive escalation and caregiver positive affect were negatively correlated with adolescent BPD symptoms. Caregiver negative affect was associated with caregiver BPD symptoms. Caregiver depressive symptoms were positively associated with negative affect and negative extrinsic emotion regulation and negatively associated with dyadic positive escalation and caregiver positive affect. Participants’ depressive symptoms in childhood were not associated with any observational codes. Regarding correlations among psychopathology variables, adolescent BPD symptoms, but not adolescent MDD symptoms, correlated positively with caregiver MDD and BPD symptoms.

**Table 3:** Bivariate correlations of study variables

	Female sex	White race	Age at last wave	BPFS-C total	MDD core	CG PAI-BOR total	CG BDI-II total	Childhood MDD core	CG positive affect	CG negative affect	CG neg extrinsic ER	Dyadic role confusion	Dyadic pos escalation	Dyadic neg escalation
Female sex	1.000													
White race	.02	1.000												
Age at last wave	.07	.09	1.000											
BPFS-C total	.13	-.16^c^	.19^c^	1.000										
MDD core	.17^c^	.03	-.01	.51^a^	1.000									
CG PAI-BOR total	-.03	-.41^a^	-.06	.26^b^	.14	1.000								
CG BDI-II total	-.00	-.19^b^	-.06	.21^b^	.13	.57^a^	1.000							
Childhood MDD core*	-.06	-.18^c^	.14	.37^a^	.33^a^	.39^a^	.47^a^	1.000						
CG positive affect	.04	.17^c^	.14	-.17^c^	-.12	-.13	-.28^a^	-.18^c^	1.000					
CG negative affect	-.04	-.27^a^	-.10	.00	.05	.25^b^	.22^b^	.29^b^	-.34^a^	1.000				
CG neg extrinsic ER	-.05	-.18^b^	-.13	-.09	-.02	.16	.24^b^	.28^b^	-.26^a^	.68^a^	1.000			
Dyadic role confusion	.12	-.11	.20^c^	-.05	-.12	.07	.06	.02	.09	.13^c^	.06	1.000		
Dyadic pos escalation	.08	.06	.03	-.30^b^	-.13	-.04	-.15^c^	-.08	.47^a^	-.12	-.08	.24^b^	1.000	
Dyadic neg escalation	-.11	-.05	.06	.010	.10	.04	.03	.13	-.09	.38^a^	.24^b^	.20^b^	.07	1.000

^a^*p* < 0.0001; ^b^*p* < 0.01; ^c^*p* < 0.05; *Childhood MDD core = mean of MDD core scores at waves 1–8; CG = Caregiver; ER = emotion regulation

Our stepwise approach revealed recurring patterns in caregiver psychopathology predicting adolescent psychopathology. The addition of caregiver BPD symptoms to regressions predicting adolescent BPD symptoms predicted larger increases in R2 than did caregiver MDD symptoms when adolescent MDD symptoms were the outcome. Caregiver MDD symptoms accounted for little R2 change in adolescent BPD symptoms, as did caregiver BPD symptoms in predicting adolescent MDD symptoms. Finally, childhood MDD was consistently predictive of both adolescent MDD and BPD in the full models (i.e., Step 4).

As detailed in Table 4, lower dyadic positive escalation was the only Hot Topics code significantly associated with later BPD severity (Est = − 8.118, t = − 4.00, FDR p < 0.001). The finding held when covarying for caregiver BPD, caregiver MDD severity, biological sex, prior depressive symptoms for the adolescent, and after false discovery rate correction. Dyadic positive escalation was also associated with later MDD severity (Est = − 0.871, t = − 2.03, *p* = 0.04), but this finding did not hold after FDR correction or when caregiver MDD and caregiver BPD severity were added as covariates (Table 5). As detailed in Table 5, no other codes were associated with MDD severity.

**Table 4:** Hierarchical linear regressions of adolescent BPD severity by hot topics codes and caregiver BPD and depression severity

Caregiver positive affect						
Step 1; R^2^ = 0.015	**Est**	**SE**	**β**	**t**	**p**	**FDR p**
Caregiver positive affect	−2.79	1.81	−0.12	−1.54	0.125	0.375
Step 2; R^2^ = 0.089, ΔR^2^ = 0.075	**Est**	**SE**	**β**	**t**	**p**	**FDR p**
Caregiver PAI-BOR total score	0.35	0.10	0.28	3.60	< 0.001	
Caregiver positive affect	−1.82	1.77	−0.08	−1.03	0.306	0.458
Step 3; R^2^ = 0.096, ΔR^2^ = 0.007	**Est**	**SE**	**β**	**t**	**p**	**FDR p**
Caregiver BDI-II total score	0.15	0.14	0.11	1.09	0.276	
Caregiver PAI-BOR total score	0.27	0.12	0.22	2.31	0.022	
Caregiver positive affect	−1.23	1.85	−0.05	−0.66	0.508	0.610
Step 4; R^2^ = 0.177, ΔR^2^ = 0.081	**Est**	**SE**	**β**	**t**	**p**	**FDR p**
Female sex	3.45	2.09	0.12	1.65	0.101	
Childhood MDD core score	2.24	0.61	0.31	3.68	< 0.001	
Caregiver BDI-II total score	−0.01	0.14	−0.01	−0.06	0.949	
Caregiver PAI-BOR total score	0.21	0.11	0.16	1.79	0.075	
Caregiver positive affect	−0.77	1.78	−0.03	−0.43	0.665	0.665
Caregiver negative affect						
Step 1; R^2^ = 0.004	**Est**	**SE**	**β**	**t**	**p**	**FDR p**
Caregiver negative affect	1.82	2.41	0.06	0.76	0.451	0.541
Step 2; R^2^ = 0.083, ΔR^2^ = 0.080	**Est**	**SE**	**β**	**t**	**p**	**FDR p**
Caregiver PAI-BOR total score	0.37	0.10	0.29	3.71	< 0.001	
Caregiver negative affect	−0.38	2.39	−0.01	−0.16	0.876	0.876
Step 3; R^2^ = 0.095, ΔR^2^ = 0.012	**Est**	**SE**	**β**	**t**	**p**	**FDR p**
Caregiver BDI-II total score	0.19	0.14	0.14	1.42	0.157	
Caregiver PAI-BOR total score	0.27	0.12	0.22	2.31	0.022	
Caregiver negative affect	−1.19	2.46	−0.04	−0.49	0.628	0.628
Step 4; R^2^ = 0.180, ΔR^2^ = 0.086	**Est**	**SE**	**β**	**t**	**p**	**FDR p**
Female sex	3.35	2.09	0.12	1.60	0.111	
Childhood MDD core score	2.33	0.61	0.32	3.81	< 0.001	
Caregiver BDI-II total score	0.03	0.14	0.02	0.22	0.823	
Caregiver PAI-BOR total score	0.21	0.11	0.17	1.81	0.072	
Caregiver negative affect	−2.23	2.39	−0.07	−0.93	0.352	0.422
Caregiver negative extrinsic emotion regulation						
Step 1; R^2^ = 0.000	**Est**	**SE**	**β**	**t**	**p**	**FDR p**
Caregiver negative extrinsic ER	0.13	2.00	0.01	0.06	0.949	0.949
Step 2; R^2^ = 0.085, ΔR^2^ = 0.085	**Est**	**SE**	**β**	**t**	**p**	**FDR p**
Caregiver PAI-BOR total score	0.37	0.10	0.30	3.83	< 0.001	
Caregiver negative extrinsic ER	−1.09	1.94	−0.04	−0.56	0.576	0.692
Step 3; R^2^ = 0.099, ΔR^2^ = 0.014	**Est**	**SE**	**β**	**t**	**p**	**FDR p**
Caregiver BDI-II total score	0.21	0.14	0.15	1.54	0.126	
Caregiver PAI-BOR total score	0.27	0.12	0.21	2.28	0.024	
Caregiver negative extrinsic ER	−1.86	2.00	−0.07	−0.93	0.354	0.532
Step 4; R^2^ = 0.188, ΔR^2^ = 0.090	**Est**	**SE**	**β**	**t**	**p**	**FDR p**
Female sex	3.34	2.07	0.12	1.61	0.109	
Childhood MDD core score	2.41	0.61	0.33	3.93	< 0.001	
Caregiver BDI-II total score	0.05	0.14	0.03	0.35	0.730	
Caregiver PAI-BOR total score	0.20	0.11	0.16	1.72	0.088	
Caregiver negative extrinsic ER	−2.97	1.94	−0.12	−1.53	0.128	0.195
Dyadic role confusion						
Step 1; R^2^ = 0.008	**Est**	**SE**	**β**	**t**	**p**	**FDR p**
Dyadic role confusion	−2.93	2.64	−0.09	−1.11	0.269	0.537
Step 2; R^2^ = 0.096, ΔR^2^ = 0.089	**Est**	**SE**	**β**	**t**	**p**	**FDR p**
Caregiver PAI-BOR total score	0.37	0.10	0.30	3.93	< 0.001	
Dyadic role confusion	−3.84	2.54	−0.12	−1.51	0.132	0.397
Step 3; R^2^ = 0.108, ΔR^2^ = 0.012	**Est**	**SE**	**β**	**t**	**p**	**FDR p**
Caregiver BDI-II total score	0.19	0.13	0.14	1.45	0.148	
Caregiver PAI-BOR total score	0.28	0.12	0.22	2.36	0.020	
Dyadic role confusion	−4.08	2.54	−0.12	−1.61	0.110	0.330
Step 4; R^2^ = 0.193, ΔR^2^ = 0.084	**Est**	**SE**	**β**	**t**	**p**	**FDR p**
Female sex	4.01	2.08	0.14	1.93	0.056	
Childhood MDD core score	2.22	0.60	0.31	3.69	< 0.001	
Caregiver BDI-II total score	0.02	0.13	0.02	0.16	0.873	
Caregiver PAI-BOR total score	0.21	0.11	0.17	1.86	0.065	
Dyadic role confusion	−4.39	2.45	−0.13	−1.79	0.076	0.195
Dyadic positive escalation						
Step 1; R^2^ = 0.094	**Est**	**SE**	**β**	**t**	**p**	**FDR p**
Dyadic positive escalation	−8.85	2.18	−0.31	−4.06	< 0.001	< 0.001
Step 2; R^2^ = 0.168, ΔR^2^ = 0.074	**Est**	**SE**	**β**	**t**	**p**	**FDR p**
Caregiver PAI-BOR total score	0.34	0.09	0.27	3.76	< 0.001	
Dyadic positive escalation	−8.44	2.10	−0.29	−4.02	< 0.001	< 0.001
Step 3; R^2^ = 0.172, ΔR^2^ = 0.003	**Est**	**SE**	**β**	**t**	**p**	**FDR p**
Caregiver BDI-II total score	0.10	0.13	0.07	0.81	0.417	
Caregiver PAI-BOR total score	0.29	0.11	0.23	2.56	0.011	
Dyadic positive escalation	−8.18	2.12	−0.28	−3.85	< 0.001	0.001
Step 4; R^2^ = 0.253, ΔR^2^ = 0.081	**Est**	**SE**	**β**	**t**	**p**	**FDR p**
Female sex	3.73	1.99	0.13	1.88	0.062	
Childhood MDD core score	2.20	0.58	0.30	3.80	< 0.001	
Caregiver BDI-II total score	−0.06	0.13	−0.05	−0.49	0.622	
Caregiver PAI-BOR total score	0.22	0.11	0.180	2.05	0.042	
Dyadic positive escalation	−8.12	2.03	−0.28	−4.00	< 0.001	< 0.001
Dyadic negative escalation						
Step 1; R^2^ = 0.004	**Est**	**SE**	**β**	**t**	**p**	**FDR p**
Dyadic negative escalation	−2.22	2.85	−0.06	−0.78	0.438	0.541
Step 2; R^2^ = 0.090, ΔR^2^ = 0.086	**Est**	**SE**	**β**	**t**	**p**	**FDR p**
Caregiver PAI-BOR total score	0.37	0.10	0.30	3.87	< 0.001	
Dyadic negative escalation	−3.02	2.74	−0.08	−1.10	0.273	0.458
Step 3; R^2^ = 0.102, ΔR^2^ = 0.012	**Est**	**SE**	**β**	**t**	**p**	**FDR p**
Caregiver BDI-II total score	0.19	0.13	0.14	1.47	0.145	
Caregiver PAI-BOR total score	0.27	0.12	0.22	2.30	0.023	
Dyadic negative escalation	−3.41	2.74	−0.10	−1.24	0.216	0.431
Step 4; R^2^ = 0.188, ΔR^2^ = 0.086	**Est**	**SE**	**β**	**t**	**p**	**FDR p**
Female sex	3.35	2.07	0.12	1.61	0.108	
Childhood MDD core score	2.32	0.61	0.32	3.84	< 0.001	
Caregiver BDI-II total score	0.02	0.13	0.02	0.16	0.873	
Caregiver PAI-BOR total score	0.20	0.11	0.16	1.77	0.079	
Dyadic negative escalation	−4.02	2.64	−0.11	−1.52	0.130	0.195

**Table 5:** Hierarchical linear regressions of adolescent MDD severity by hot topics codes and caregiver depression and BPD severity

Caregiver positive affect						
Step 1; R^2^ = 0.017	**Est**	**SE**	**β**	**t**	**p**	**FDR p**
Caregiver positive affect	-0.58	0.34	-0.13	-1.68	0.095	0.286
Step 2; R^2^ = 0.036, ΔR^2^ = 0.019	**Est**	**SE**	**β**	**t**	**p**	**FDR p**
Caregiver BDI-II total score	0.04	0.02	0.15	1.76	0.081	
Caregiver positive affect	-0.37	0.36	-0.09	-1.03	0.306	0.611
Step 3; R^2^ = 0.039, ΔR^2^ = 0.003	**Est**	**SE**	**β**	**t**	**p**	**FDR p**
Caregiver PAI-BOR total score	0.02	0.02	0.06	0.67	0.504	
Caregiver BDI-II total score	0.03	0.03	0.11	1.05	0.296	
Caregiver positive affect	-0.38	0.36	-0.09	-1.06	0.292	0.584
Step 4; R^2^ = 0.145, ΔR^2^ = 0.106	**Est**	**SE**	**β**	**t**	**p**	**FDR p**
Female sex	0.95	0.40	0.18	2.36	0.020	
Childhood MDD core score	0.46	0.12	0.33	3.91	< 0.001	
Caregiver PAI-BOR total score	0.00	0.02	0.01	0.08	0.936	
Caregiver BDI-II total score	-0.01	0.03	-0.02	-0.19	0.850	
Caregiver positive affect	-0.28	0.34	-0.06	-0.80	0.422	0.781
Caregiver negative affect						
Step 1; R^2^ = 0.011	**Est**	**SE**	**β**	**t**	**p**	**FDR p**
Caregiver negative affect	0.61	0.46	0.11	1.34	0.181	0.363
Step 2; R^2^ = 0.032, ΔR^2^ = 0.021	**Est**	**SE**	**β**	**t**	**p**	**FDR p**
Caregiver BDI-II total score	0.04	0.02	0.16	1.86	0.064	
Caregiver negative affect	0.32	0.48	0.06	0.66	0.508	0.762
**Step 3; R**^**2**^** = 0.035, ΔR**^**2**^** = 0.002**	**Est**	**SE**	**β**	**t**	**p**	**FDR p**
Caregiver PAI-BOR total score	0.01	0.02	0.06	0.57	0.568	
Caregiver BDI-II total score	0.03	0.03	0.12	1.23	0.219	
Caregiver negative affect	0.30	0.48	0.05	0.62	0.536	0.782
**Step 4; R**^**2**^** = 0.142, ΔR**^**2**^** = 0.107**	**Est**	**SE**	**β**	**t**	**p**	**FDR p**
Female sex	0.98	0.40	0.18	2.42	0.017	
Childhood MDD core score	0.46	0.12	0.33	3.86	< 0.001	
Caregiver PAI-BOR total score	0.00	0.02	0.00	0.02	0.982	
Caregiver BDI-II total score	-0.00	0.03	-0.00	-0.03	0.976	
Caregiver negative affect	0.13	0.46	0.02	0.28	0.781	0.781
Caregiver negative extrinsic emotion regulation						
Step 1; R^2^ = 0.003	**Est**	**SE**	**β**	**t**	**p**	**FDR p**
Caregiver negative extrinsic ER	0.28	0.38	0.06	0.75	0.456	0.475
Step 2; R^2^ = 0.030, ΔR^2^ = 0.026	**Est**	**SE**	**β**	**t**	**p**	**FDR p**
Caregiver BDI-II total score	0.05	0.02	0.17	2.07	0.040	
Caregiver negative extrinsic ER	0.04	0.39	0.01	0.11	0.914	0.914
Step 3; R^2^ = 0.032, ΔR^2^ = 0.002	**Est**	**SE**	**β**	**t**	**p**	**FDR p**
Caregiver PAI-BOR total score	0.01	0.02	0.06	0.62	0.537	
Caregiver BDI-II total score	0.04	0.03	0.14	1.34	0.181	
Caregiver negative extrinsic ER	0.05	0.39	0.01	0.12	0.908	0.908
Step 4; R^2^ = 0.142, ΔR^2^ = 0.110	**Est**	**SE**	**β**	**t**	**p**	**FDR p**
Female sex	0.96	0.40	0.18	2.38	0.018	
Childhood MDD core score	0.47	0.12	0.34	3.96	< 0.001	
Caregiver PAI-BOR total score	0.00	0.02	0.00	0.02	0.985	
Caregiver BDI-II total score	0.00	0.03	0.01	0.10	0.920	
Caregiver negative extrinsic ER	-0.15	0.38	-0.03	-0.41	0.685	0.781
Dyadic role confusion						
Step 1; R^2^ = 0.007	**Est**	**SE**	**β**	**t**	**p**	**FDR p**
Dyadic role confusion	-0.51	0.50	-0.08	-1.02	0.309	0.464
Step 2; R^2^ = 0.040, ΔR^2^ = 0.033	**Est**	**SE**	**β**	**t**	**p**	**FDR p**
Caregiver BDI-II total score	0.05	0.02	0.18	2.34	0.021	
Dyadic role confusion	-0.63	0.50	-0.10	-1.27	0.205	0.611
Step 3; R^2^ = 0.042, ΔR^2^ = 0.003	**Est**	**SE**	**β**	**t**	**p**	**FDR p**
Caregiver PAI-BOR total score	0.02	0.02	0.06	0.67	0.505	
Caregiver BDI-II total score	0.04	0.03	0.15	1.50	0.135	
Dyadic role confusion	-0.65	0.50	-0.10	-1.29	0.197	0.584
Step 4; R^2^ = 0.155, ΔR^2^ = 0.112	**Est**	**SE**	**β**	**t**	**p**	**FDR p**
Female sex	1.06	0.40	0.20	2.62	0.010	
Childhood MDD core score	0.46	0.12	0.33	3.93	< 0.001	
Caregiver PAI-BOR total score	0.00	0.02	0.01	0.10	0.918	
Caregiver BDI-II total score	0.00	0.03	0.01	0.12	0.907	
Dyadic role confusion	-0.75	0.48	-0.12	-1.57	0.119	0.356
Dyadic positive escalation						
Step 1; R^2^ = 0.025	**Est**	**SE**	**β**	**t**	**p**	**FDR p**
Dyadic positive escalation	-0.87	0.43	-0.16	-2.03	0.044	0.262
Step 2; R^2^ = 0.048, ΔR^2^ = 0.023	**Est**	**SE**	**β**	**t**	**p**	**FDR p**
Caregiver BDI-II total score	0.04	0.02	0.15	1.93	0.055	
Dyadic positive escalation	-0.75	0.43	-0.14	-1.73	0.085	0.509
Step 3; R^2^ = 0.051, ΔR^2^ = 0.003	**Est**	**SE**	**β**	**t**	**p**	**FDR p**
Caregiver PAI-BOR total score	0.02	0.02	0.07	0.70	0.483	
Caregiver BDI-II total score	0.03	0.03	0.11	1.15	0.252	
Dyadic positive escalation	-0.76	0.43	-0.14	-1.76	0.080	0.480
Step 4; R^2^ = 0.160, ΔR^2^ = 0.109	**Est**	**SE**	**β**	**t**	**p**	**FDR p**
Female sex	0.99	0.40	0.18	2.49	0.014	
Childhood MDD core score	0.46	0.12	0.33	3.95	< 0.001	
Caregiver PAI-BOR total score	0.00	0.02	0.01	0.13	0.899	
Caregiver BDI-II total score	-0.01	0.03	-0.02	-0.23	0.818	
Dyadic positive escalation	-0.752	0.41	-0.14	-1.84	0.067	0.356
Dyadic negative escalation						
Step 1; R^2^ = 0.003	**Est**	**SE**	**β**	**t**	**p**	**FDR p**
Dyadic negative escalation	0.39	0.54	0.06	0.72	0.475	0.475
Step 2; R^2^ = 0.031, ΔR^2^ = 0.028	**Est**	**SE**	**β**	**t**	**p**	**FDR p**
Caregiver BDI-II total score	0.05	0.02	0.17	2.13	0.035	
Dyadic negative escalation	0.25	0.54	0.04	0.46	0.649	0.779
Step 3; R^2^ = 0.033, ΔR^2^ = 0.002	**Est**	**SE**	**β**	**t**	**p**	**FDR p**
Caregiver PAI-BOR total score	0.01	0.02	0.06	0.62	0.539	
Caregiver BDI-II total score	0.04	0.03	0.13	1.37	0.174	
Dyadic negative escalation	0.24	0.54	0.04	0.45	0.652	0.782
Step 4; R^2^ = 0.142, ΔR^2^ = 0.108	**Est**	**SE**	**β**	**t**	**p**	**FDR p**
Female sex	0.98	0.40	0.18	2.42	0.017	
Childhood MDD core score	0.46	0.12	0.34	3.91	< 0.001	
Caregiver PAI-BOR total score	0.00	0.02	0.00	0.04	0.969	
Caregiver BDI-II total score	0.00	0.03	0.00	0.00	0.999	
Dyadic negative escalation	0.15	0.51	0.02	0.29	0.775	0.781

## Discussion

This study examined whether caregiver and dyadic behaviors in late childhood and early adolescence differentially predicted BPD vs. MDD symptomatology in late adolescence, contributing to recent efforts to identify developmental factors that differentially predict one psychiatric disorder from the other (e.g., Huntington et al., 2025). Across models, caregiver psychopathology was associated with subsequent adolescent psychopathology, but positive escalation between child and caregiver was the only caregiving or dyadic behavior that uniquely predicted subsequent adolescent psychopathology – in this case, BPD, but not MDD. The association was negative, such that higher levels of positive escalation during childhood and early adolescent conflict discussions predicted lower BPD symptomatology in late adolescence.

The caregiving and dyadic behaviors included in our analyses were selected because of their theoretical and empirically-demonstrated connections with BPD and/or MDD symptoms; for example, parental negative affect and negative escalation in the parent–child dyad are known correlates of offspring with depressive and BPD symptoms (Carlson et al., 2009; Lyons-Ruth et al., 2015; Whalen et al., 2014; Yap et al., 2011). In this sample, however, when controlling for caregivers’ MDD and BPD symptoms, only one observed behavior (i.e., positive escalation) emerged as a significant predictor of child symptoms. Parental psychopathology shapes parent–child and parenting behaviors (Berg-Nielsen et al., 2002) and so may share much of the variance with parenting and parent–child behaviors. The caregiver and caregiver-child behaviors we assessed for in our study may represent just a small subset of the ways caregiver psychopathology influences child psychopathology; other mechanisms, such as modeling of individual and dyadic behaviors which the child observes but does not participate in (Breaux et al., 2016); other forms of emotion socialization; or broader stress exposure, might more strongly predict child psychopathology outcomes.

Our finding found that dyadic positive escalation negatively predicted BPD severity, even after controlling for caregiver BPD and MDD symptoms and child MDD symptoms, is consistent with prior work; Whalen and colleagues (2014) observed decreases in BPD severity over time for children who were high in positive escalation during a conflict discussion with their mothers, but not for children in dyads with less positive escalation. Interpreted through the lens of biosocial theory (Linehan, 1993), positive escalation during a conflict discussion might reflect validation, rather than invalidation, of the child’s emotional experience. This exchange of positive affect, especially in the context of a conflict discussion, may allow more opportunity and permission for a child to voice their needs and negatively-valanced emotions (Schofield et al., 2012). Positive escalation in this context may signal to the child that the full range of their emotions are welcome and they are still perceived positively, even in emotionally charged situations, protecting against the relational discord and upheaval that are risk factors for BPD. In this way, feeling connected and safe with a caregiver in a challenging conversation could promote a positive sense of self and protect against the development of the incoherent or negative self-concept associated with BPD (Stanley & Siever, 2010).

Although prior research has documented lower rates of positive interactions in dyads featuring a depressed child and/or mother (McMakin et al., 2011; Sheeber et al., 2009), positive escalation in conflict discussions did not predict adolescent MDD in our study after correcting for multiple comparisons and adjusting for caregiver psychopathology and having oversampled for depressive symptoms in the first place. Other parent–child interactions involving negative affect, such as parental reinforcement of negative emotions, may exert a stronger influence on the development of depressive symptoms than interactions involving positive affect. An alternative explanation is that low levels of positive affect are so characteristic of families with depression that controlling for parental depression masks any effect of the parent–child dynamic.

More broadly, research on the developmental course of depression does not always consider comorbidity with BPD and other personality pathology, and many of the identified developmental risk factors for BPD also predict risk for multiple other psychiatric disorders, such as depression (Stepp et al., 2016). In our analyses, which are among the few to include both caregiver BPD and MDD as predictors of offspring BPD and MDD, caregiver depressive symptoms often approached significance in predicting subsequent adolescent BPD symptoms, even when controlling for caregiver BPD. While these results are not statistically significant and should not be over-interpreted, they underscore the value of considering both domains of parental psychopathology when studying the developmental pathways to either personality pathology or depressive disorders. Future research should consider how each type of parental psychopathology might make unique contributions to risk for offspring BPD.

Clinically, our findings highlight the value of reciprocally positive interactions between caregiver and child as a treatment target in psychotherapy and other clinical interventions. Dialectical behavioral therapy (DBT), the gold standard approach to treating BPD, can incorporate family members in treatment (e.g., Mehlum et al., 2014; Uliaszek et al., 2014); participants in one DBT study perceived increased parental positivity to be an especially important outcome of such treatment (Rathus et al., 2015). The specific context of our findings – a conflict discussion – should be taken into consideration here: specific instruction and support in remaining positive during challenging conversations might be most effective in buffering against BPD risk. Relatedly, clinicians could assess for the presence or absence of positive escalation between caregiver and child, as it may be a risk factor for subsequent BPD risk. The case for intervention around positive escalation could be strengthened by connecting positive escalation as observed in family therapy sessions, or through passive smartphone data collection (e.g., analyzing text exchanges), to youth BPD features. Another, more direction for subsequent research would be characterizing on a more granular level conversations that do and do not feature positive escalation, to better understand, for example, specific contributions made by the child or the caregiver to initiating or sustaining positive escalation.

### Strengths and Limitations

Regarding limitations to this study, first, participants in this study were oversampled for preschool depressive symptoms; although we controlled for earlier depressive symptoms in our sample, our results may not generalize to a broader population less characterized by early psychopathology. Second, we collected data on adolescent BPD symptoms through self-report, rather than the gold standard approach of clinical interviewing, which was used for MDD symptoms. Individuals with BPD may demonstrate a slight response bias in self-reporting (e.g., tending away from midpoint responding; Kaufman et al., 2023), which could be implicated in our significant association of BPD symptoms with positive escalation. Thus, replication with a clinical interview measure of BPD would strengthen the case for this association. Third, we may not be able to detect smaller effects in the data due to our relatively small sample size. In addition, several interaction codes were infrequently coded and required dichotomization, which may reduce sensitivity to more nuanced variation in dyadic behavior. Finally, our childhood MDD score had relatively low internal consistency across the eight time points it was collected; this may limit our ability to accurately understand the role of childhood MDD symptoms in our analyses.

In terms of strengths, our study is among the very few to leverage longitudinal observational data to relate caregiving and caregiver-child behaviors to subsequent child psychopathology, let alone BPD specifically. We also used rigorous controls (i.e., caregiver BPD and MDD symptoms) and corrected for the false discovery rate, creating a high threshold in terms of predictive power for our caregiving and caregiver-child behaviors. Finally, we drew attention to the value of including measures of both caregiver personality pathology and mood symptoms as predictors of child personality pathology and psychopathology.

## Conclusion

In this study, we drew on prior observational research with parent–child dyads to present the first attempt to longitudinally and simultaneously predict adolescent BPD and MDD symptoms from caregiving and caregiver-child behaviors at earlier developmental stages. After controlling for caregiver BPD and MDD and correcting for multiple comparisons, dyadic positive escalation between caregiver and child negatively predicted BPD symptoms in late adolescence. Our findings contribute to the nascent literature on differential developmental predictors of BPD versus MDD risk and highlight positive escalation as a potential treatment target for psychotherapeutic approaches to BPD.

## Data Availability

Data may available from the first author upon request.
